# The dental perspective on osteogenesis imperfecta in a Danish adult population

**DOI:** 10.1186/s12903-018-0639-7

**Published:** 2018-10-24

**Authors:** Kirstine Juhl Thuesen, Hans Gjørup, Jannie Dahl Hald, Malene Schmidt, Torben Harsløf, Bente Langdahl, Dorte Haubek

**Affiliations:** 1 Section for Pediatric Dentistry, Department of Dentistry and Oral Health, Health, Aarhus University, Vennelyst Boulevard 9, 8000 Aarhus C, Denmark; 2 Center for Oral Health in Rare Diseases, Department of Maxillofacial Surgery, Aarhus University Hospital, Noerrebrogade 44, 8000 Aarhus C, Denmark; 3 Department of Endocrinology and Internal Medicine, Aarhus University Hospital, Tage-Hansens Gade 2, Aarhus, Denmark; 4 Aarhus Municipal Dental Service, Groendalsvej 2, 8000 Aarhus C, Denmark

**Keywords:** Collagen, Dentinogenesis imperfecta, Dental treatment load, Radiographic findings

## Abstract

**Background:**

To report on dental characteristics and treatment load in Danish adult patients with osteogenesis imperfecta (OI).

**Methods:**

Oral examination of 73 patients with OI was performed and OI type I, III, and IV were represented by 75.3%, 8.2%, and 16.4%, respectively. Patients were diagnosed as having dentinogenesis imperfecta (DI) if they had clinical and radiological signs of DI. In the data analysis, mild OI (type I) was compared to moderate-severe OI (type III and IV).

**Results:**

Discoloration of teeth was prevalent in patients with moderate-severe compared to mild OI (83.3% vs. 5.5%, *p* < 0.001). Cervical constriction and pulpal obliteration were frequent findings in patients with moderate-severe OI (61.1% and 88.9%, respectively), whereas pulp stones and taurodontism were diagnosed in patients with mild OI only (29.1% and 9.1%, respectively). DI was found in 24.7% of OI patients and considerably more frequent in patients with moderate-severe (94.4%) compared to mild OI (1.8%) (*p* < 0.001). The number of teeth with artificial crowns was significantly higher in patients with moderate-severe OI than in patients with mild OI (median 1.5, range 0–23 vs. median 0, range 0–14) (*p* < 0.001). The number of teeth with fillings in patients with mild OI was significantly higher than in patients with moderate-severe OI (mean 9.7, SD 5.1, median 9.0, range 1–21 vs. mean 5.0, SD 4.4, median 4.0, range 0–16) (*p* < 0.001).

**Conclusions:**

One fourth of patients with OI had DI, and the vast majority of them had moderate-severe OI. Whereas discoloration of teeth, cervical constriction and pulp obliteration were frequent findings in patients with moderate-severe OI, pulp stones and taurodontism were found in patients with mild OI only. In patients with moderate-severe OI, the dental treatment load was dominated by prosthetic treatment, whereas restorative treatment with fillings was more prevalent in patients with mild OI.

## Background

Osteogenesis imperfecta (OI) is a rare hereditary connective tissue disorder with different degrees of severity. The prevalence of OI in Denmark is estimated to 11 per 100.000 [1]. According to Sillence [2], type I is classified as a phenotype with low degree of deformity and near to normal stature, type II is the most severe form with perinatal death, type III is the most severe type with high degree of deformity and a very short stature, and type IV is the moderately affected phenotype with a severity between OI type I and III. The majority of OI cases are caused by autosomal dominant mutations in one of the two genes, *COL1A1* and *COL1A2* [3–5] that encode the α1(I) and the α2(I) chains of collagen type I, which is an important component in bone and dentine. Collagen mutations can lead to either quantitative or qualitative collagen abnormality, i.e., a reduced amount of structurally normal collagen respective the formation of an abnormally structured collagen [6].

In addition to OI, some patients have dentinogenesis imperfecta (DI) which is a hereditary dentine disorder characterized by greyish-blue to brown discoloration of teeth and pulp obliteration [7]. DI is currently subdivided into three subtypes. DI type I is associated with OI and is caused by mutations in the genes, *COL1A1* and *COL1A2* encoding collagen type I. DI type II and III are caused by mutations in the gene encoding dentine sialophosphoprotein (*DSPP*) [7], and not found in OI patients. In DI, the enamel appears normal in structure, but is vulnerable due to the underlying abnormal dentine. Structurally, dentine is composed of hydroxyapatite, an organic phase composed primarily of type I collagen and water. Due to mutations in the genes encoding collagen type I, defects in the dentine occur [8]. In patients with DI type I, both dentitions are typically affected, but the primary dentition is usually more severely affected than the permanent dentition [9].

The aim of the present study was to report on dental characteristics and treatment load in adult Danish patients with OI.

## Methods

### Study population

The present study is based on a dental investigation of 73 patients with genetically-verified OI, recruited in a cross-sectional clinical study originally based on 85 Danish adult patients with OI [10]. With the overall aim to investigate medical aspects of OI in adult patients, the inclusion criteria were clinically confirmed diagnosis of OI and an age ≥ 18 years. Exclusion criteria were comorbidity of cancer, treatment with glucocorticoids equivalent to 5 mg prednisolone or more within the last 3 months, other metabolic bone diseases, renal diseases, or hepatic diseases. The final diagnosis of OI was based on consensus between three investigators (JDH, TH, and BL). The classification of OI in subtypes was based on criteria by Sillence and coworkers [2]. The recruitment of patients took place from 2010 to 2013 through databases at university hospitals and regional hospitals in Denmark and by advertisements in the Journal of the Danish OI Patient Society, DFOI.

Among the 85 patients with OI included in the clinical study, two were excluded due to lack of teeth, and ten did not participate for various reasons, leaving 73 participants for the dental assessment. Among the 73 participants, 63 (86.3%) had a skin biopsy taken during the medical study or previously. Skin biopsy was not obtained in 10 patients, either because they were unwilling to undergo the procedure, due to infection in the laboratory, or the procedure was not possible for logistic reasons [11].

### Clinical and radiological dental examination

A dental examination was performed at Department of Dentistry and Oral Health, Aarhus University, including the recording of the teeth present in the oral cavity and the previous dental treatment carried out. Fillings in teeth were recorded as well as artificial crowns, bridges, and the use of removable dentures. As a part of the investigation, clinical photos were obtained. A full-mouth periapical survey with digital intraoral radiographs, using GX 1000 dental X-ray© (Gendex, Des Plaines, IL, USA) as well as a digital panoramic radiograph, using the digital radiographic equipment Planmeca Promax© (Planmeca Oy, Helsinki, Finland), were obtained.

In the evaluation of the dental hard tissues, an assessment of the radiographs included: The number of teeth present, signs of obliterated pulp chamber, presence of pulp stone, short root, cervical constriction, and the dental treatment load. The recorded findings were obtained by a consensus among three co-authors (DH, HG, and KJT) after assessing clinical recordings, radiological signs, and evaluation of clinical photos.

The clinical signs of DI are increased translucency of enamel and greyish-blue to brown discoloration of teeth. In addition, abnormal radiographic signs like large pulp chambers in teeth under development and eruption, early and advanced or total pulp obliteration of fully developed teeth, short roots, cervical constriction, pulp stones, and taurodontism have been reported [12–15].

### Statistics

In the data analysis, the mild OI (type I) was compared to moderate-severe OI (type III and IV). Descriptive statistics were used to summarize all data. Differences according to the severity of OI were evaluated by students t-test or Fischer’s exact test as appropriate. Data analysis was performed using STATA 11.0 (StataCorp, College Station, Texas). In the present study, *p*-values < 0.050 were considered statistically significant.

## Results

Seventy three patients (mean age: 45.4 years (SD 14.5)) with OI underwent a clinical and radiological dental examination, meaning 85.9% of the original clinical study population. According to OI severity, 55 (75.3%) patients had mild OI (type I) and 18 (24.7%) patients had moderate-severe OI (6 OI type III and 12 OI type IV). The group of 73 patients consisted of 53.4% women and 46.6% men (*p* = 0.424). According to OI severity, the mean age of patients with mild OI was 46.2 years (SD 2.1) and 42.9 years (SD 2.4) for the patients with moderate-severe OI (*p* = 0.400).

The quantitative collagen defect was associated with DI in only one out of 38 patients, all having mild OI, and the qualitative collagen defect was associated with DI in 17 out of 18 patients, all having moderate-severe OI (*p* < 0.001) (Table 1).

**Table 1:** OI severity and the presence of DI according to collagen I abnormality

		Collagen types			
		Normal	Quantitative^a^	Qualitative^b^	Unknown
OI severity	Presence of DI				
Mild	÷ DI	3	37	4	10
	+ DI	0	1	0	0
Moderate-severe	÷ DI	0	0	1	0
	+ DI	0	0	17	0

^a^Deviation in the amount of collagen I in skin biopsy

^b^Deviation in the structure of collagen I in skin biopsy

**Fig. 1:**
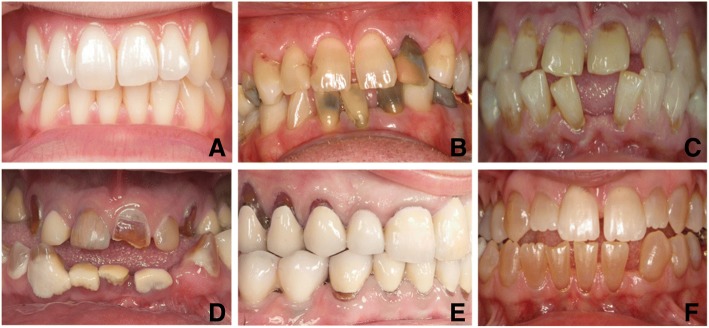
The variation of the dental view of patients with OI. **a** OI type I without DI. **b** OI type I without DI, discoloration induced by endodontic therapy. **c** OI type III without DI. **d** OI type III with DI, including dental calculus accumulation. **e** OI type IV with DI, treated with artificial crowns. **f** OI type IV with DI

**Fig. 2:**
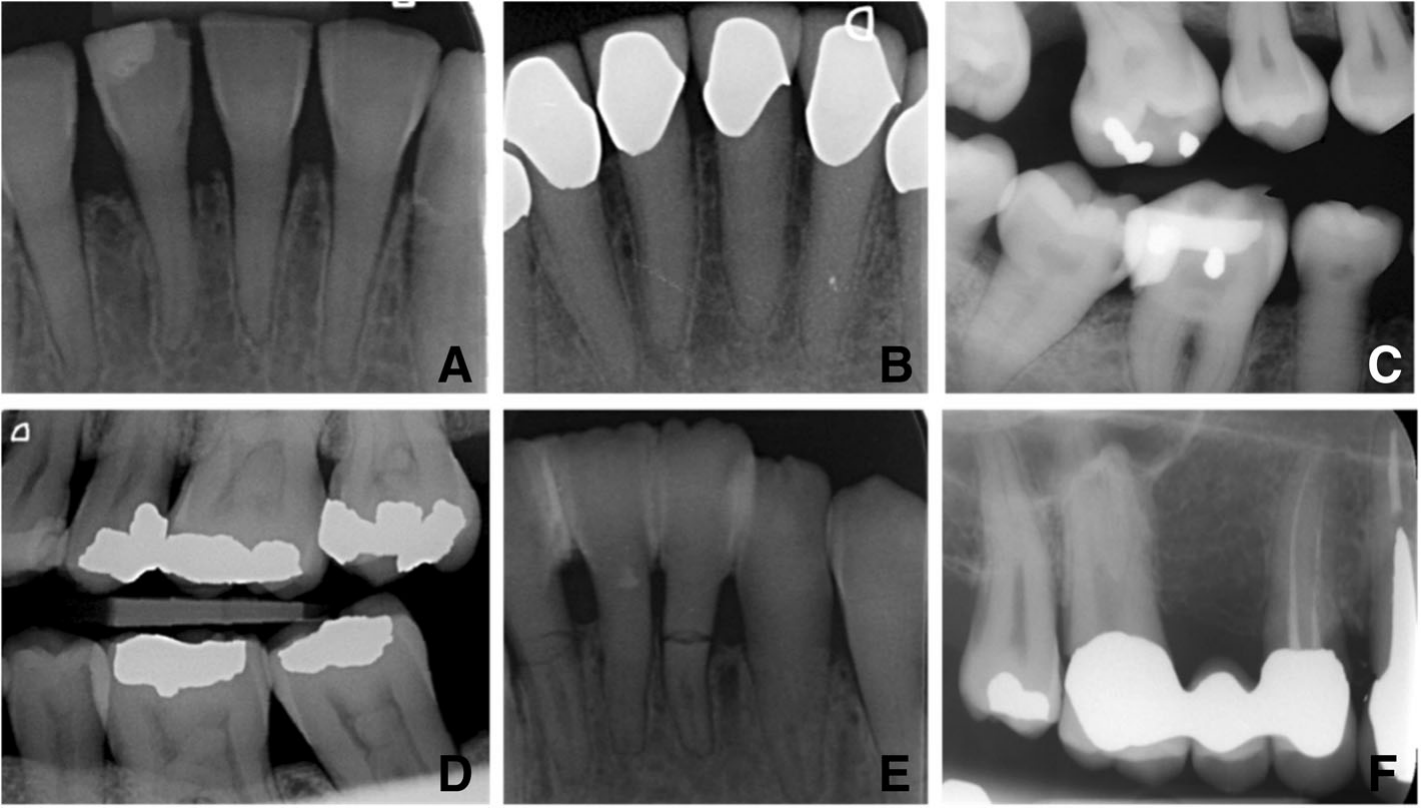
The variation of radiographic signs of DI as seen on dental radiographs of patients with OI. **a** OI type IV with DI, incisor with short root and pulp obliteration. **b** OI type IV with DI, incisor with pulp obliteration and treated with artificial crowns. **c** OI type III with DI, molars and premolars with cervical constriction. **d** OI type I, pulp stones in 26, 27, 36 and 37. **e** OI type III with DI, incisor with pulp obliteration and root fractures. **f** OI type I, taurodontism

The distribution of patients with OI according to OI severity and dental characteristics is presented in Table 2. Discoloration of teeth, either as greyish or brownish color, was found more often in patients with moderate-severe OI than in patients with mild OI (15 out of 18 (83.3%) vs. 3 out of 55 (5.5%)) (*p* < 0.001) (Table 2, Fig. 1, d and f), and also pulp obliteration (16 out of 18 (88.9%) vs. 4 out of 55 (7.3%) (*p* < 0.001)) and short roots (9 out of 18 (50%) vs. 4 out of 55 (7.3%) (*p* < 0.001)) appeared more often in patients with moderate-severe OI than in patients with mild OI (Fig. 2, a, b and e). Cervical constriction appeared in patients with moderate-severe OI only (11 out of 18 (61.1%) patients) (*p* < 0.001) (Table 2, Fig. 2, c). In contrast, pulp stones and taurodontism were found in patients with mild OI only (16 out of 55 (29.1%) (*p* = 0.008) and 5 out of 55 (9.1%) (*p* = 0.322), respectively) (Table 2, Fig. 2, d and f).

**Table 2:** Dental characteristics in 73 patients with OI

OI severity	Mild		Moderate-Severe		
	OI type I	OI type III	OI type IV	OI type III and IV	
	(*n* = 55)	(*n* = 6)	(*n* = 12)	(*n* = 18)	P*
Diagnosed with DI (%)	1 (1.8)	5 (83.3)	12 (100)	17 (94.4)	< 0.001
Discoloration of teeth (%)^a^	3 (5.5)	5 (83.3)	10 (83.3)	15 (83.3)	< 0.001
Pulp obliteration (%)	4 (7.3)	4 (66.7)	12 (100)	16 (88.9)	< 0.001
Short root (%)	4 (7.3)	4 (66.7)	5 (41.7)	9 (50)	< 0.001
Pulp stone (%)^b^	16 (29.1)	0	0	0	0.008
Cervical constriction (%)	0	6 (100)	5 (41.7)	11 (61.1)	< 0.001
Taurodontism (%)^c^	5 (9.1)	0	0	0	0.322

The figures are number of patients with the specified type of dental characteristic, and the percentages in the respective OI severity groups are given in parentheses

*P-value according to Fischer’s exact test. OI type I (mild) compared to OI type III and IV (moderate-severe)

^a^Missing data in one patient with mild and three patients with moderate-severe OI

^b^Missing data in one patient with mild and one patient with moderate-severe OI

^c^Missing data in one patients with mild OI

Patients with both discoloration and radiological signs of DI were diagnosed as having DI, being a total of 18 of 73 (24.7%) patients with OI. Among these 18 patients with DI, 1 (1.4%) had mild OI and 17 (94.4%) had moderate-severe OI (*p* < 0.001) (Table 2).

The number of teeth present in the oral cavity of patients with OI varied from 5 to 32 teeth (mean: 25.8, SD 5.0). The mean number of teeth present in patients with mild OI was 26.0 (SD 5.5; range 5–32), and not significantly different from the mean number of teeth present in patients with moderate-severe OI (mean: 25.3, SD 3.9; range 16–32) (*p* = 0.657). The mean number of teeth present in the group of patients with DI was not significantly different to the mean number in the group of patients without DI (mean: 25.4 (SD 3.9) and mean: 25.9 (SD 5.4), respectively) (*p* = 0.686).

**Fig. 3:**
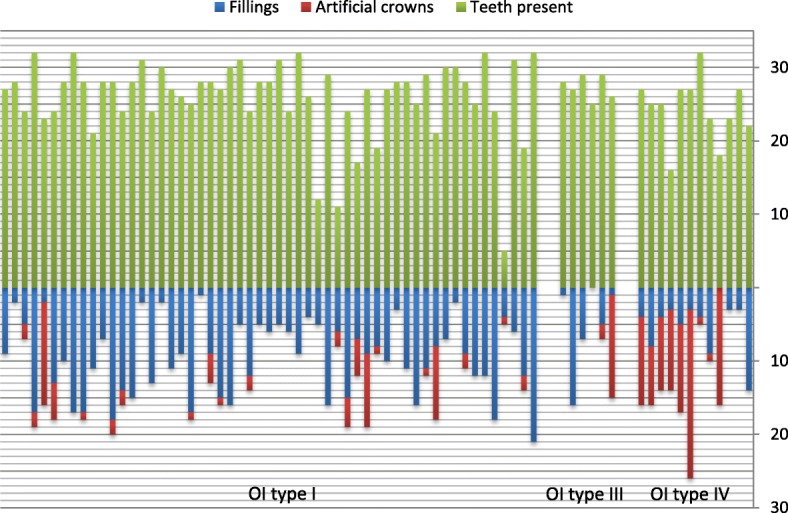
Illustration of the dental treatment load in 73 patients with OI. For the individual patient, the number of teeth present and the number of teeth treated with fillings respective artificial crowns are illustrated

The overall dental treatment load, including fillings and artificial crowns, is illustrated in Fig. 3. The number of teeth with fillings in patients with OI varied from 0 to 21 teeth and was in patients with mild OI (mean 9.7, SD 5.1, median 9.0, range 1–21) significantly higher than in patients with moderate-severe OI (mean 5.0, SD 4.4, median 4.0, range 0–16) (*p* < 0.001). The median number of filled teeth relative to the total number of teeth present was in the mild OI group 0.38 (range 0.04–0.80) and in moderate-severe OI 0.15 (range 0–0.64) (*p* = 0.001).

Thirty-two out of 73 (43.8%) patients with OI were treated with artificial crowns with the median number of crowns being 2.0 (range 1–23). Eleven out of 18 (61.1%) patients with moderate-severe OI had teeth treated with artificial crowns compared to 21 out of 55 (38.2%) patients with mild OI (*p* = 0.107). The median number of teeth with artificial crowns in patients with moderate-severe OI was 1.5 (range 0–23) and was 0 (range 0–14) in patients with mild OI (*p* < 0.001). The median number of teeth with artificial crowns relative to the total number of teeth present in mild OI group was 0 (range 0–0.6) and in the moderate-severe OI group 0.01 (range 0–0.9) (*p* = 0.001).

In total, eight patients had removable dentures (Table 3). In the mild OI group, two patients had a complete maxillary denture and one of these also had a partial mandibular denture. In addition, five patients with mild OI and one patient with moderate-severe OI had a removable partial denture.

**Table 3:** The type of prosthetic treatment in 73 patients with OI

OI severity	Mild		Moderate-Severe		
	OI type I	OI type III	OI type IV	OI type III and IV	
	(*n* = 55)	(*n* = 6)	(*n* = 12)	(*n* = 18)	P*
Artificial crown (%)	21 (38.2)	2 (33.3)	9 (75)	11 (61.1)	0.107
Tooth-supported bridge (%)	6 (10.9)	0	3 (25)	3 (16.7)	0.680
Implant (%)	2 (3.6)	0	4 (33.3)	4 (22.2)	0.030
Implant-supported bridge (%)	1 (1.8)	0	2 (16.7)	2 (11.1)	0.435
Removable denture (%)	7 (12.7)	0	1 (8.3)	1 (5.6)	0.435

The figures are number of patients with the specified type of treatment, and the percentages in the respective OI severity groups are given in parentheses

*P-value according to Fischer’s exact test. OI type I (mild) compared to OI type III and IV (moderate-severe)

## Discussion

The present cross-sectional study shows that DI is mainly found in the patients with qualitative collagen defects and moderate-severe OI and rarely seen in patients with quantitative defects and mild OI. Prosthetic treatment with artificial crowns and fixed partial dentures was carried out more often in the group of patients with moderate-severe OI compared to patients with mild OI. In the latter group of patients, conventional fillings and removable dentures were predominant.

A cross-sectional design was chosen in the present study. Although it has limitations, this type of study design is useful in studies on small populations, characterized by a rare disease, as it adds to the existing knowledge of the rare disease more than case reports do. The present study aimed at including a representative sample of adults with OI living in Denmark [10]. The majority of adult OI patients are routinely followed in Denmark on one of the three university hospitals in Aarhus, Odense or Hvidovre. Participants were recruited by advertisements in the Danish OI magazine, through regional hospitals, databases and Center for Rare Diseases, leading to the participation of both patients with mild and moderate-severe OI. A high participation rate of patients with mild OI was found (75.3%). In a previous Danish study by Lund and coworkers (1998), the participants were all referred patients to the Department of Clinical Genetics, Rigshospitalet, Copenhagen, Denmark, thus a relative high frequency of patients with moderate-severe OI (43.2%) was included in that study [14]. The broad representation of all OI types, including a high number of patients with mild OI, is a strength of the present study. Though, concerning the representativity of the study population, some severely affected patients might having been unable to travel for the participation in the investigation, but on the other hand some mildly affected patients might having been too busy to participate in a time-consuming investigation. Thus, the study population is likely to reflect the relative distribution of OI types.

The dental characteristics of OI patients were assessed based on clinical examination, clinical photos and radiographs. Due to physical proportions, shortness of the neck and immobility, some patients with OI were challenging to examine. On the other hand, OI patients were very cooperative and willing to take part in the procedures needed to establish the diagnostic material collected in the present study.

The frequency of obliterated pulp, short roots, pulp stone, cervical constriction and taurodontism in various populations and patients groups varies when searching through the literature. No validated methods, based on specific definitions for diagnosing dental findings as obliterated pulp, pulp stones, and cervical constriction are available, except for taurodontism [16]. Thus, the overall radiographic diagnosis was based on visual assessment of the radiographs, and reached after consensus between the involved coworkers.

As both Saeves and coworkers [12] and Malmgren and coworkers [13] point out, normally colored teeth and absence of radiographic signs of DI do not necessarily indicate the absence of dentin abnormalities, which might be found if a histological examination was included. This is clearly described by Andersson and coworkers [15]. Hence, the diagnosis of DI may vary according to the parameters and tests on which the diagnosis is based. It is well-known that the number of tests, on which a diagnosis is based, is affecting the number of patients found. On the other hand, the assessment of dental agenesis on radiographs is, for example, a valid method. But in the present study population on adults, which included elderly individuals with a highly reduced number of teeth, our information on the reason(s) for the absence of teeth was sparse. Thus, dental agenesis was not assessed in the present study.

DI was diagnosed in 24.7% of the patients with OI. This corresponds roughly to the previous Danish study by Lund and coworkers [14] who reported the prevalence of DI to be 28% of the study group and a recent Swedish study by Andersson and coworkers [15], who reported the prevalence of DI to be 29% when based on a clinical and radiographic diagnostics. In a Norwegian study by Saeves and coworkers [12], the frequency of DI was 19%. The Norwegian study group consisted of 94 participants aged 25 years or older. In contrast, the appearance of DI was reported to be 42% in the Swedish study by Malmgren and coworkers [13] and raised to 48% in the above-mentioned Swedish study when histological assessment was included [15]. However, all patients in the Swedish studies were referred to a specialized diagnostic unit, thus may have included more patients with moderate-severe types of OI. The mild OI group in the two Swedish studies had a high DI prevalence (28% and 31%, respectively) compared to the present study (1.8%). Major differences in the composition of the various study populations mentioned above, and the recruitment of them, are likely to explain the differences between the results obtained. Furthermore, the patients of the Swedish studies were aged 0.3 to 20 years. In the present Danish study, the patients were adults, and consequently all had permanent dentition. Malmgren and coworkers point out that in the mixed dentition the permanent teeth were less affected than the primary teeth in terms of both discoloration and attrition [13]. Thus, the inclusion of children may explain the higher prevalence of DI in some previous studies [13–15] compared to the present and the Norwegian studies on adults only [12]. In this context, it is, however, an important point that the thickness of enamel of permanent teeth is twice that of primary teeth. Dentine dysplasia is visible through translucent enamel. Although not real, DI in the permanent dentition may ‘seem milder’ than in the primary dentition.

In the present study, pulp stones were found in patients with mild OI only (16 out of 55 (29.1%)) (Table 2). Contrary, pulp stones were most frequently found in patients with moderate-severe OI in the study by Lund and coworkers (moderate-severe OI: 10 out of 12 (83%); mild OI: 18 out of 30 (60%)) [14]. In the Swedish study by Malmgren and coworkers [13], pulp stones were rare (4.2%). But the presence of artificial crowns complicates the possibility of detecting pulp stones on radiographs. As patients with moderate-severe OI often are characterized by DI and treatment with artificial crowns, this may explain the finding of no pulp stones in patients with moderate-severe OI in our study. Furthermore, the physiological obliteration of the pulp by age is ‘accelerated’ in patients with moderate-severe OI, and by age this phenomenon might gradually blur the possibility of diagnosing pulp stones. In addition, pulp stones are prevalent in the normal population (20.7%) [17], and this proportion is similar to the findings in patients with OI type I in the present study.

An evaluation of the dental treatment load according to OI severity showed an obvious tendency toward significantly more advanced treatment, such as fixed prosthetics, carried out in the group of moderate-severe OI patients compared to patients with mild OI, where conventional fillings and removable dentures were the predominant treatment types preformed. As the majority of patients with moderate-severe OI also had DI, it is likely that DI is the main reason for the more comprehensive use of fixed prosthetics. The health care system in Denmark gives patients with dental anomalies, like DI, the possibility to receive financial support when they are in need of dental treatment. This regulation might explain the more advanced and expensive treatments performed in patients with moderate-severe OI. In contrast, treatment with conventional fillings because of caries is only minimally supported by the health care system.

In the study by Saeves and coworkers [12], they found that the mean number of filled teeth was 13.5 in OI type I, and 11.0 in type III and IV. It is mentioned that some patients also had implants and artificial crowns carried out, but detailed information on the extent is not provided. In the present study, the mean number of filled teeth was 9.7 in OI type I, and 5.0 for type III and IV. The mean number of teeth with artificial crowns was 1.4 in OI type I, and 6.1 for type III and IV. In addition, implants were present in one third of patients with OI type III and IV in contrast to 5% in patients with type I. According to O’Connel and Marini, theoretically the binding of resin may be compromised, but clinically it appears successful [9]. Thus, composite fillings can be made also in patients with OI if enamel is present. The difference in treatment load may therefore be because of prevalent crown fractures and decay in teeth affected by DI, but might also be influenced by the previously mentioned possibility for governmental financial support when teeth are affected by DI. The relatively high number of fillings and low number of crowns in the mild OI group without DI can be explained by either less severe decay of the dentition or by patient’s omission of the expensive prosthetic treatment options, which is without governmental financial support, when teeth are not affected by DI. These questions remain unanswered by the present cross-sectional study.

## Conclusions

One fourth of patients with OI had DI, and the vast majority of them had moderate-severe OI. Whereas discoloration of teeth, cervical constriction and pulp obliteration were frequent findings in patients with moderate-severe OI, pulp stones, and taurodontism were found in patients with mild OI only. In patients with moderate-severe OI, the dental treatment load was dominated by prosthetic treatment including implant-based prostheses, whereas restorative treatment with fillings was more prevalent in patients with mild OI. Governmental financial support seems to facilitate the choice of prosthetic treatment in patients with DI.

## Abbreviations

*COL1a1*: Gene encoding collagen type 1a1
*COL1a2*: Gene encoding collagen type 1a2
DI: Dentinogenesis imperfecta
*DSPP*: Gene encoding dentine sialophosphoprotein
Fig.: Fig.
OI: Osteogenesis imperfecta
p: *P*-value according to Fischer’s exact test
SD: Standard deviation
vs.: Versus
